# Hsp40 Protein DNAJB6 Interacts with Viral NS3 and Inhibits the Replication of the Japanese Encephalitis Virus

**DOI:** 10.3390/ijms20225719

**Published:** 2019-11-14

**Authors:** Yu-Qin Cao, Lei Yuan, Qin Zhao, Jian-Lin Yuan, Chang Miao, Yung-Fu Chang, Xin-Tian Wen, Rui Wu, Xiao-Bo Huang, Yi-Ping Wen, Qi-Gui Yan, Yong Huang, Xin-Feng Han, Xiao-Ping Ma, San-Jie Cao

**Affiliations:** 1 Research Center of Swine Disease, College of Veterinary Medicine, Sichuan Agricultural University, Chengdu 611130, China; b20162007@stu.sicau.edu.cn (Y.-Q.C.); zhao.qin@sicau.edu.cn (Q.Z.); 2018103011@stu.sicau.edu.cn (J.-L.Y.); mcdragon@sicau.edu.cn (C.M.); xintian@sicau.edu.cn (X.-T.W.); wurui1977@sicau.edu.cn (R.W.); huangxiaobo@sicau.edu.cn (X.-B.H.); wyp@sicau.edu.cn (Y.-P.W.); yqg@sicau.edu.cn (Q.-G.Y.); hyong601@sicau.edu.cn (Y.H.); hanxinf@sicau.edu.cn (X.-F.H.); mxp886@sicau.edu.cn (X.-P.M.); 2 Sichuan Science-observation Experiment of Veterinary Drugs and Veterinary Diagnostic Technology, Ministry of Agriculture, Chengdu 611130, China; 3 Pathogen and Immunology Experiment Teaching Center, North Sichuan Medical College, Nanchong 637100, Sichuan, China; b20172403@stu.sicau.edu.cn; 4 Department of Population Medicine and Diagnostic Sciences, College of Veterinary Medicine, Cornell University, Ithaca, NY 14850, USA; yc42@cornell.edu; 5 National Teaching and Experiment Center of Animal, Sichuan Agricultural University, Chengdu 611130, Sichuan, China

**Keywords:** flavivirus, Japanese encephalitis virus, non-structural protein 3 (NS3), the DnaJ heat shock protein family (Hsp40) member B6 (DNAJB6), replication, virus–host interactions

## Abstract

The Japanese encephalitis virus (JEV) is a mosquito-borne flavivirus prevalent in east and southeast Asia, the Western Pacific, and northern Australia. Since viruses are obligatory intracellular pathogens, the dynamic processes of viral entry, replication, and assembly are dependent on numerous host-pathogen interactions. Efforts to identify JEV-interacting host factors are ongoing because their identification and characterization remain incomplete. Three enzymatic activities of flavivirus non-structural protein 3 (NS3), including serine protease, RNA helicase, and triphosphatase, play major roles in the flaviviruses lifecycle. To identify cellular factors that interact with NS3, we screened a human brain cDNA library using a yeast two-hybrid assay, and identified eight proteins that putatively interact with NS3: COPS5, FBLN5, PPP2CB, CRBN, DNAJB6, UBE2N, ZNF350, and GPR137B. We demonstrated that the DnaJ heat shock protein family (Hsp40) member B6 (DNAJB6) colocalizes and interacts with NS3, and has a negative regulatory function in JEV replication. We also show that loss of DNAJB6 function results in significantly increased viral replication, but does not affect viral binding or internalization. Moreover, the time-course of DNAJB6 disruption during JEV infection varies in a viral load-dependent manner, suggesting that JEV targets this host chaperone protein for viral benefit. Deciphering the modes of NS3-interacting host proteins functions in virion production will shed light on JEV pathogenic mechanisms and may also reveal new avenues for antiviral therapeutics.

## 1. Introduction

Viruses of the Flaviviridae family are responsible for significant morbidity and mortality, and are increasing in prevalence worldwide. Mosquito-borne diseases caused by flaviviruses have spread rapidly in recent years. The prevalence of Dengue, West Nile, and Japanese encephalitis, three major mosquito-borne viruses, are an expanding public health threat. These pathogens currently threaten the health of millions of individuals, and are a potential threat in non-endemic areas that are colonized by mosquitos [1]. The tremendous global, social, and economic impact associated with the diseases caused by these viruses necessitate strong therapeutic intervention [2].

The Japanese encephalitis virus (JEV), a member of the genus Flavivirus, has the potential to become a global pathogen. JEV has five genotypes (I, II, III, IV, and V), Genotype III has been predominant since the 1950s, and vaccines of JEV genotype III have been used worldwide for vaccination against JEV infection [3]. However, recently, JEV genotype I has been circulating widely in some Asian countries; thus, the efficiency of the present Japanese encephalitis (JE) vaccine in eliciting protective neutralizing antibodies against different JEV strains needs to be evaluated [4]. In addition, JE has no specific treatment, except a few supportive treatments. Japanese encephalitis (JE) is a severe viral encephalitis that is prevalent in Asia (especially the rural areas of eastern and southeastern Asia), the Western Pacific, and northern Australia [5]. Although 67,900 JE cases have been estimated among 24 JE epidemic countries annually, the reported JE cases are decreasing due to the establishment of JE surveillance and vaccine in some countries. However, serious outbreaks still occur [6]. With the rise in global temperatures, and the increased range of competent mosquitoes, the spread of JEV to Europe and North America appears probable [7]. Since viruses are obligatory intracellular pathogens, an in-depth understanding of the mechanisms of the replication cycle and the interactions with host organisms are required to develop strategies to combat their infection [8].

JEV is a single-stranded, positive-sense RNA virus that encodes a polyprotein that is co- and post-translationally cleaved into 10 proteins by viral and host proteases. The structural proteins, capsid (C), precursor membrane (prM/M), and envelope (E) are integrated into the virion. The seven non-structural proteins, NS1, NS2A, NS2B, NS3, NS4A, NS4B, and NS5 coordinate different intracellular processes, including virus replication, assembly, and release. Some non-structural proteins also modulate host defense mechanisms and antagonize the host immune response. JEV NS5 inhibits the induction of type I IFN by interacting with KPNA3 and KPNA4 [9], the NS5 protein of various flaviviruses has developed distinct mechanisms to antagonize different steps of the IFN-α/β signaling [10]. NS3 and NS5 are the main enzymatic components of the viral replication complex and are crucial to the life cycle of flavivirus [11].

Although viruses do encode for proteins to perform functions for their propagation, they are totally reliant on their hosts [12]. They are obligate intracellular parasites that utilize host translation machinery to ensure efficient production of their progeny; at the same time, host cells have developed strategies to combat their infection. To achieve an effective infection, viruses need to find the proper balance between their dependency on the host cell translation machinery and potentially adverse effects of antiviral proteins [13]. Modulation of host antiviral responses, by targeting the cellular proteins and cofactors required for the viral life cycle, represents a major strategy to restrict viruses’ propagation and spread [14]. Considerable progress has been made elucidating JEV structure and replication strategies, but the complex molecular interactions between the virus and host remain obscure, in contrast to the more in depth knowledge of Japanese encephalitis virus receptors [15].

In recent years, a growing number of studies have identified several host factors associated with JEV. Nain et al. identified GRP78 interacting with recombinant JEV envelope protein domain III and plays a dual role in virus entry and replication [16]. Chiu et al. demonstrated that ZAP may act as an intrinsic antiviral factor through specific RNA binding to fight against JEV infection [17]. Ma et al. demonstrated that SPCS1 affects viral replication by interacting with transmembrane domains of NS2B [18]. Niu et al. demonstrated that TIM-1 is associated with JEV susceptibility to host cells; TIM-1 promotes JEV infection as an entry cofactor [19]. Additionally, JEV infection is also regulated by microRNAs. Hazra et al. showed that the host microRNA miR-301a blocks the IRF1-mediated neuronal innate immune response to JEV infection [20]. Ashraf et al. demonstrated that miR-19b-3p positively regulates the JEV-induced inflammatory response via targeting RNF11 [21].

Non-structural protein 3 (NS3) is one of the best characterized non-structural proteins of JEV; it is a multifunctional protein with three distinct activities: serine protease, RNA helicase, and triphosphatase. The serine protease is required for cleavage of the polyprotein, the helicase/NTPase is required for unwinding the double-stranded replicative form of RNA, and RNA triphosphatase is needed for capping nascent viral RNA [22,23]. In addition to its enzymatic roles, NS3 also plays non-enzymatic roles in autophagy, fatty acid biosynthesis, and actin polymerization, mediated by its recruitment of host proteins from a variety of cellular pathways [24]. Together with the viral RNA, viral cofactors, and host cell cofactors, NS3 acts as a hub, and in close association with NS5 forms the viral replication complex which assembles on the intracellular membrane [24,25].

Although most studies of JEV NS3 focus on its protease, helicase, and NTPase activities, some research has begun to elucidate the JEV NS3 protein interactome leading to the identification of many proteins that interact with NS3. Le Breton et al. performed a high-throughput yeast two-hybrid screen to identify the interactions between human host proteins and the flavivirus NS3 and NS5 proteins, and identified 108 human proteins interacting with NS3 or NS5 proteins or both [11]. Chiou et al. proved that microtubules and TSG101 associate with JEV NS3, which is incorporated into the JEV-induced structure during JEV replication [26]. Hsp70 is associated with the components of viral replicase complex including NS3, NS5 during JEV infection [27]. EEF1A1 can stabilize the components of viral replicase complex by interacting with the JEV proteins NS3 and NS5 and thus facilitates viral replication [28]. However, the information on NS3-targeting proteins and their impact on virus replication is still limited.

In this study, yeast two-hybrid screening and co-immunoprecipitation were performed to identify novel host proteins that interact with NS3. We demonstrated for the first time that the DnaJ heat shock protein family (Hsp40) member B6 (DNAJB6) interacts and colocalizes with NS3. Functional analysis showed that overexpression of DNAJB6 restricted JEV propagation, while in DNAJB6-deficient cells JEV propagation was significantly increased. Exogenous addition of DNAJB6 to DANJB6 deficient cells partially counteracted the effect. The loss of DNAJB6 function did not affect JEV binding to or entry into cells. Our results indicate that DNAJB6 participates in viral replication, but is not involved in the entry stage of JEV infection.

## 2. Results

### 2.1. Screening for Cellular Factors That Interact with JEV NS3

**Figure 1:**
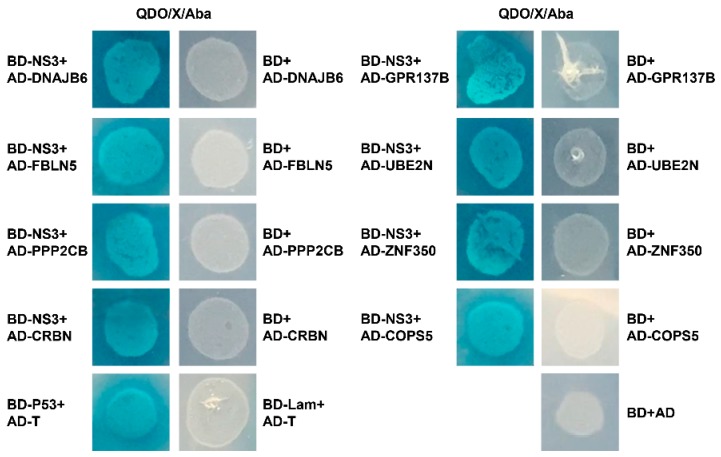
A yeast two-hybrid screen of a human brain cDNA library reveals NS3-interacting host factors. The yeast strain Y2HGold was co-transformed with the prey plasmid AD-host factors and the bait plasmid BD or BD-NS3. Co-transformation with BD/AD, BD-Lamin/AD-T, and BD-p53/AD-T were used as blank, negative, and positive controls, respectively. The screen yielded eight host factors that potentially interact with NS3.

To better understand the role of NS3 in the JEV lifecycle, and to identify cellular factors that interact with NS3 and potentially affect viral propagation, we conducted a yeast two-hybrid screen of a human brain cDNA library, using full-length NS3 as bait. After three rounds of screening, positive clones were acquired and sequenced. Sequence analysis revealed eight cellular proteins that potentially interact with JEV NS3 (COPS5, FBLN5, PPP2CB, CRBN, DNAJB6, UBE2N, ZNF350, and GPR137B; Table 1). The interactions between the eight proteins and NS3 were verified in a second round of screens in which the candidate plasmids were paired individually with the NS3 bait plasmid. The eight candidates were all confirmed to interact with JEV NS3 protein, while the negative, blank, and activation controls were all negative (Figure 1).

### 2.2. DNAJB6 Co-Precipitates and Co-Localizes with JEV NS3

The results of yeast two-hybrid screening indicated that DNAJB6 interacts with JEV NS3. DNAJB6, a member of the DnaJ/Hsp40 family, has been shown to play a role in the life cycle of HIV-1 [29,30,31,32], RSV [30] HIV-2 [33], and Human Cytomegalovirus [34], and more recently has been reported to be involved in dengue virus [35] infection. In addition, several studies have shown that Hsp70s serve as putative JEV receptors and play a vital role in JEV lifecycle [15,16,36,37,38,39,40]. DNAJB6 was chosen for further study as it is a member of the DnaJ/Hsp40 family, which is a crucial partner for Hsp70 chaperones. A large proportion of the functional diversity of Hsp70s is driven by a diverse class of cofactors, the J-proteins (also called Hsp40s) [41,42].

**Figure 2:**
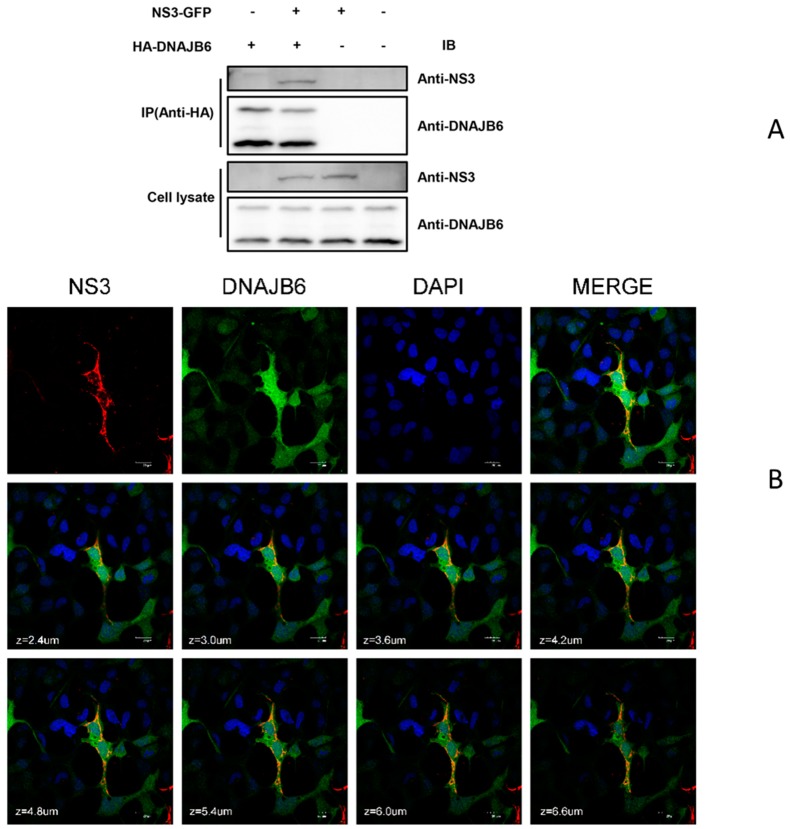
NS3-DNAJB6 interaction verified by co-immunoprecipitation and confocal immunofluorescence microscopy. (**A**) HEK293T cells were co-transfected with a GFP-tagged NS3 expression plasmid with or without HA-DNAJB6 for 48 h. Cell lysates were immunoprecipitated with anti-HA antibody. The immunoprecipitates and whole cell lysates were analyzed by Western blot using anti-DNAJB6 or anti-NS3. (**B**) DNAJB6 colocalizes with JEV NS3 in HEK293 cells. HEK293 cells transfected with plasmids expressing HA-DNAJB6 for 24 h were infected with JEV for another 36 h. These were subjected to immunofluorescence assay using anti-DNAJB6 and anti-NS3 antibodies, followed by Alexa 488 anti-rabbit and Alexa 555 anti-mouse antibodies. Cells were imaged on a confocal microscope. Magnification, ×600. Bars, 20μm. Z-stacks were acquired by sequential scanning. Abbreviations: IP: immunoprecipitation; IB, immunoblotting.

Co-immunoprecipitation (Co-IP) was performed to determine the specific interaction of NS3 with DNAJB6 in mammalian cells. DnaJB6 exists as two alternatively spliced isoforms: DnaJ6Ba and DnaJB6b. DnaJ6Ba is predominantly nuclear, while DnaJB6b is in the nucleus and cytoplasm [35]. As seen in Figure 2A, DNAJB6 co-immunoprecipitates with NS3 and the anti-human DNAJB6 antibody we used targets the N-terminus of the protein; thus, both isoforms are recognized. It should also be noted that the DNAJB6 bands in the negative controls of the cell lysate panel are due to endogenous DNAJB6. We next examined the intracellular localization of DNAJB6 and NS3 in JEV-infected HEK293 cells with antibodies specific for DNAJB6 and JEV NS3 using an immunofluorescence assay (Figure 2B). DNAJB6 localizes to the cytosol and nucleus, while JEV NS3 is only in the cytosol, as can be seen in the figure there is extensive colocalization of DNAJB6 and NS3 in the cytoplasm in JEV-infected cells. These results indicate that DNAJB6 interacts with JEV NS3. Figure S1A,B illustrates the specificity of the anti- DNAJB6 and JEV NS3 antibodies by immunofluorescence.

### 2.3. Overexpression of DNAJB6 Inhibits JEV Infection

**Figure 3:**
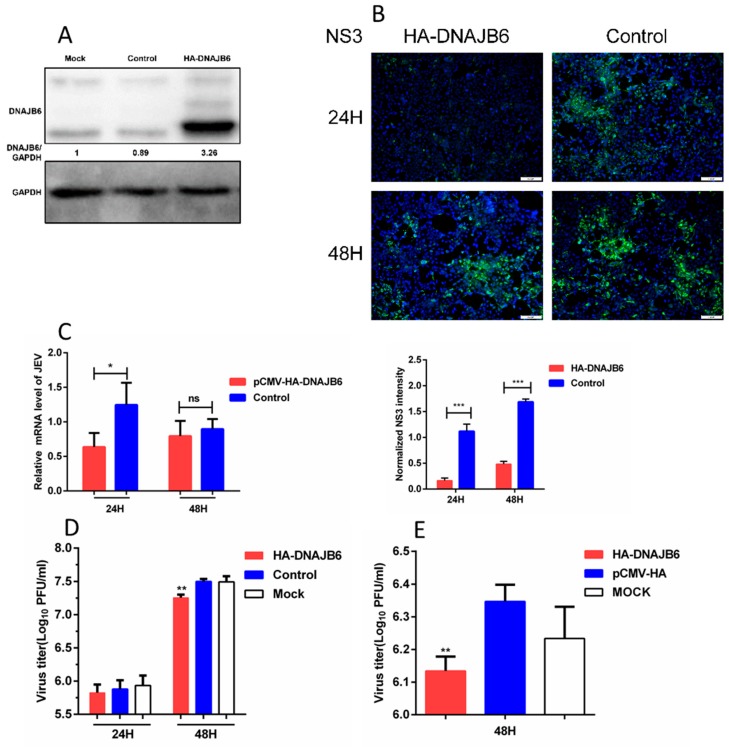
Overexpression of DNAJB6 inhibits JEV infection. (**A**) Western blot analysis of HEK293 cells overexpressing DNAJB6. The relative DNAJB6 levels from the cells before they used for subsequent experiments were all determined using Western blot analysis to ensure DNAJB6 was overexpressed. The mock (untransfected) and control (transfected with empty vector) lanes illustrate the level of endogenous expression of DNAJB6. (**B**–**D**) Infection assays of HEK293 cells overexpressing DNAJB6 then infected with JEV at MOI of 1.0 for 24 h and/or 48 h. (**B**) JEV infection measured by NS3 protein (green) immunofluorescence. Scale bar, 100 µm. Quantitation of the JEV NS3 signal integrated density was normalized to the control cells (Mean ± SD, *n* = 3, Student’s *t* test; *** *p* < 0.001). (**C**) Viral mRNA levels measured by qRT-PCR (Mean ± SD, *n* = 3, Student’s *t* test; * *p* < 0.05, ns, not significant). (**D**) JEV titers measured by plaque assay (Mean ± SD, *n* = 3, one-way ANOVA; ** *p* < 0.01). (**E**) SK-N-SH cells overexpressing DNAJB6 then infected with JEV at MOI of 1.0 for 48 h. JEV titers were determined by plaque assay (Mean ± SD, *n* = 3, Student’s *t* test; ** *p* < 0.01).

Next, utilizing HEK293 cells overexpressing DNAJB6, we set out to determine if DNAJB6 plays a role in the propagation of JEV. HA-DNAJB6 or empty plasmid were transfected into HEK293 cells followed by infection with JEV. Western blot confirmed the overexpression of DNAJB6, and the level of endogenous expression in control and mock transfected cells (Figure 3A). Immunofluorescence images of these cells after infection, probed with JEV NS3-specific rabbit polyclonal antibody, revealed that NS3 protein levels were markedly decreased in cells over-expressing DNAJB6 at 24 and 48 h post-infection (Figure 3B). JEV mRNA levels were significantly decreased in cells overexpressing DNAJB6 at 24 hpi, but there was no significant difference at 48 hpi (Figure 3C). The viral titers were lower in cells overexpressing DNAJB6 at 24 hpi, although not significantly, while they were significantly decreased at 48 hpi (Figure 3D). JEV titers were also decreased in the SK-N-SH cell line at 48 hpi (Figure 3E). Taken together, these results demonstrate that overexpression of DNAJB6 inhibits the propagation of JEV. Figure S1C,D illustrate the sensitivity of the anti-JEV NS3 antibody by Western blot and immunofluorescence, respectively.

### 2.4. Loss of DNAJB6 Function Affects the Propagation of JEV

**Figure 4:**
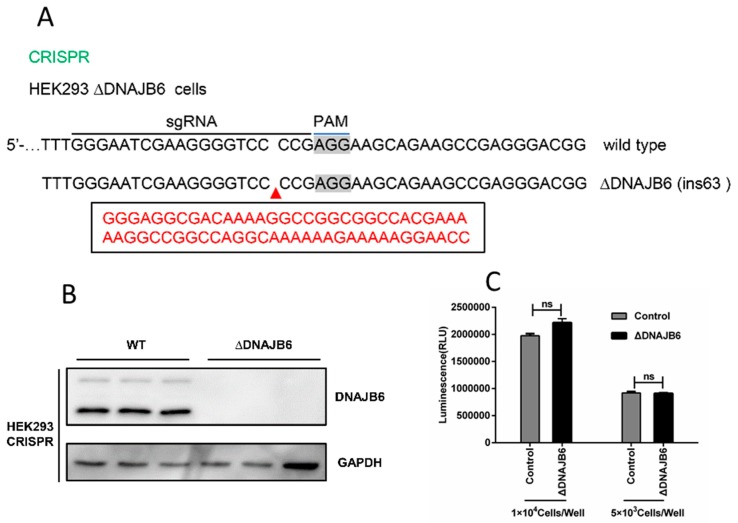
Generation and validation of DNAJB6 knockout cells. (**A**) Illustration of the disrupted alleles of DNAJB6 in HEK293 cells using CRISPR/Cas9. (**B**) DNAJB6 knockout in cell clones was verified by Western blot, wild type (WT) HEK293 cells are the control. (**C**) Cell viability assays based on quantitation of ATP. ΔDNAJB6 and parental cells were seeded at 5 × 10^3^ or 1 × 10^4^ cells per well in 96-well plates in DMEM/10% FBS. Luminescence was recorded 10 min after reagent addition. (Mean ± SD, *n* = 3, Student’s t test; ns, not significant).

Using the CRISPR/Cas9 system, we generated HEK293 cells deficient in DNAJB6 expression (Figure 4A). The lack of DNAJB6 expression was verified by Western blot (Figure 4B). Cell viability assays, based on quantitation of ATP, which signals the presence of metabolically active cells, demonstrated that the viability of the ΔDNAJB6 cells were unaffected by the deletion (Figure 4C).

**Figure 5:**
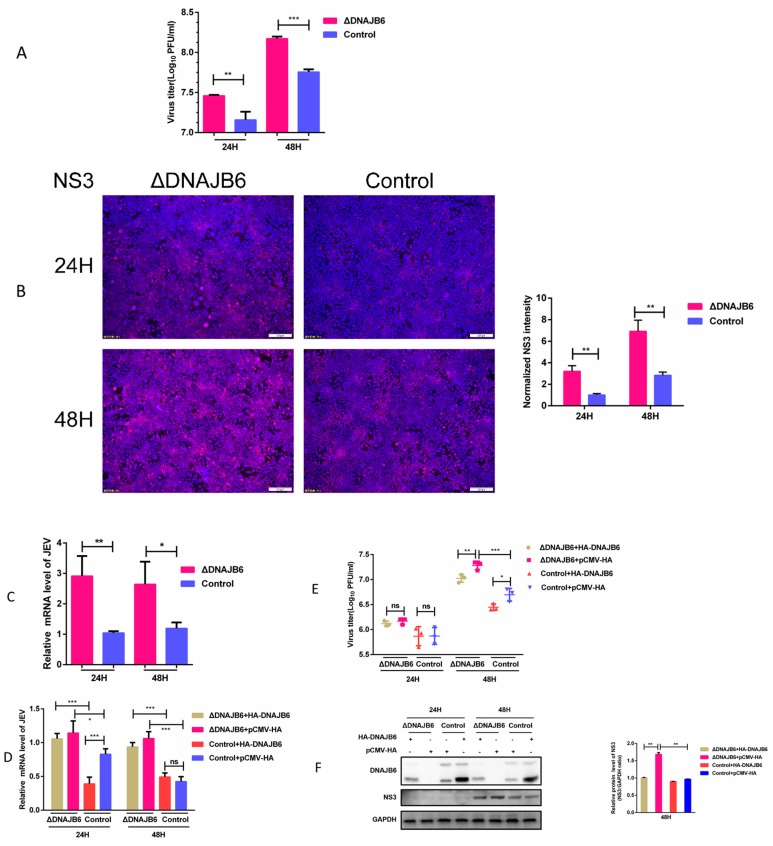
Effect of the loss of DNAJB6 on propagation of JEV. (**A**–**C**) Knocking out host factor DNAJB6 results in increased JEV propagation. ΔDNAJB6 and parental cells were infected with JEV at MOI of 1.0. At 24 and 48 hpi, JEV infection measured by (**A**) plaque assay for viral titers, (**B**) immunofluorescence for viral NS3 protein (red) expression, scale bar = 100 µm, and (**C**) qRT-PCR for viral mRNA levels. Quantitation of the NS3 signal integrated density normalized to the control is provided. (**D**–**F**) Expression of human DNAJB6 in ΔDNAJB6 cells resulted in partially restored anti-JEV activity. ΔDNAJB6 and parental cells were transfected with the DNAJB6 expressing plasmid or empty vector, followed by infection with JEV at MOI of 1.0. At 24 and 48 hpi JEV infection measured by (**D**) qRT-PCR for viral mRNA levels, (**E**) plaque assay for viral titers, and (**F**) Western blot for NS3 expression, GAPDH was used as an internal control. (Mean ± SD, *n* = 3, Student’s *t* test; * *p* < 0.05, ** *p* < 0.01, *** *p* < 0.001, ns, not significant).

ΔDNAJB6 and parental HEK-293 cells challenged with JEV were compared for efficiency of JEV propagation. The titers from ΔDNAJB6 culture supernatants were significantly higher than from parental cells (Figure 5A). Viral NS3 protein expression levels were higher in ΔDNAJB6 cells than in parental cells, as visualized by immunofluorescence microscopy (Figure 5B). It should be noted that the infectious titers from ΔDNAJB6 cells correlated well with the expression levels of NS3 in these cells. JEV mRNA levels were also significantly higher in ΔDNAJB6 cells than in parental HEK293 cells as measured by RT-qPCR (Figure 5C). These results show that the infectivity of JEV in ΔDNAJB6 cells is significantly enhanced over parental cells. We next evaluated the effect of trans-complementation of DNAJB6 on JEV propagation in ΔDNAJB6 cells. ΔDNAJB6 cells transfected with the DNAJB6 expressing plasmid had levels of JEV mRNA, NS3 expression, and viral titers, were lower than in empty vector transfected ΔDNAJB6 cells indicating expression of DNAJB6 in ΔDNAJB6 cells partially recovered virus production to levels similar to that in parental cells (Figure 5D–F). Taken together, these results demonstrate that loss of DNAJB6 is responsible for the observed increase in JEV production.

### 2.5. DNAJB6 Inhibits the Replication of JEV, but Does not Affect Viral Entry

**Figure 6:**
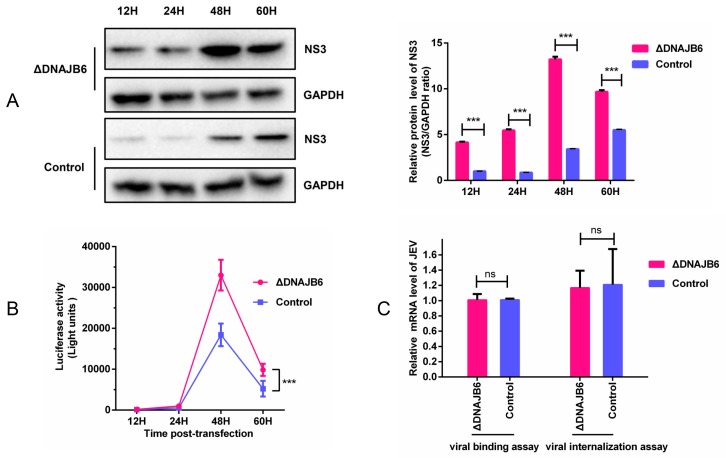
DNAJB6 inhibits JEV replication but does not affect viral entry. (**A**) Detection of the expression level of replicon NS3 in ΔDNAJB6 cells and control cells. 2 μg of in vitro-transcribed replicon RNA was transfected into 10^6^ ΔDNAJB6 and parental cells, lysates were collected at the indicated times post transfection and the NS3 expression was analyzed by Western blot. GAPDH was used as an internal control. (**B**) Luciferase activity of JEV-Rluc-Rep in ΔDNAJB6 and parental cells. Cells were treated as described above; luciferase assays were performed at the indicated time points post transfection (Mean ± SD, *n* = 3, two-way ANOVA; *** *p* < 0.001). (**C**) Role of DNAJB6 in JEV binding and internalization into cells. Virus binding to and internalization into cells were measured by qRT-PCR of JEV RNA (Mean ± SD, *n* = 3, Student’s *t* test, ns, not significant).

Viral entry and viral replication are two crucial steps of the virus life cycle. To investigate the effect of DNAJB6 on JEV replication, ΔDNAJB6 and parental HEK293 cells were transfected with the JEV subgenomic replicon SA14/U14163-Replicon (Rluc-rep). The results showed that the NS3 from the replicon was markedly increased in ΔDNAJB6 cells compared with the parental cells (Figure 6A). The absence of DNAJB6 resulted in higher Renilla luciferase activity than in the parental cells (Figure 6B). These data collectively demonstrate that DNAJB6 negatively affects JEV replication.

Because Hsp70 proteins serve as putative JEV receptors and the DnaJ/Hsp40 proteins are crucial partners for Hsp70 chaperones, we examined the role of DNAJB6 in JEV entry into cells. qRT-PCR results demonstrated that there was no significant difference between ΔDNAJB6 and parental cells in JEV RNA levels (Figure 6C), demonstrating that DNAJB6 does not affect JEV binding or entry.

### 2.6. JEV Infection Downregulates the Expression of DNAJB6

**Figure 7:**
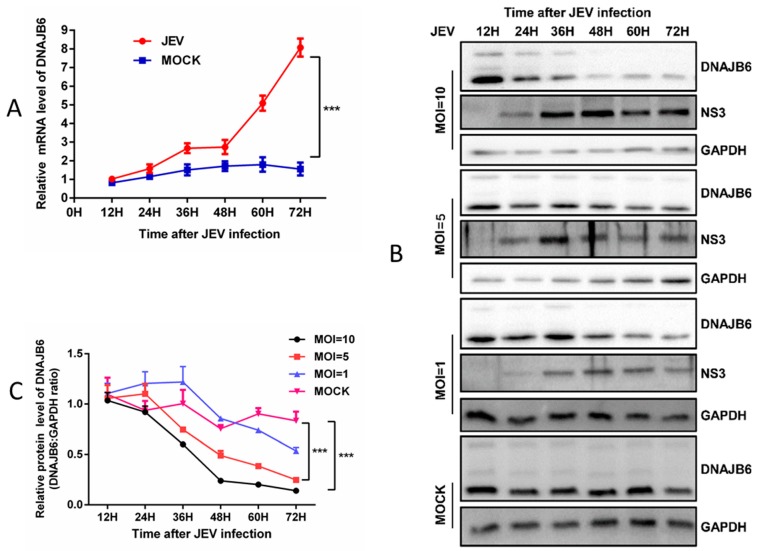
JEV infection downregulates the expression of DNAJB6. (**A**) DNAJB6 mRNA levels in mock and JEV infected (MOI of 1.0) HEK293 cells. The total cellular RNA was extracted and the levels of DNAJB6 were determined by RT-qPCR and normalized to β-actin at each time point. (B-C) DNAJB6 protein levels in mock and JEV infected HEK293 cells. (**B**) Western blot was performed to examine the expression of the cellular DNAJB6 protein, GAPDH was used as a loading control. (**C**) Quantitation of DNAJB6 protein normalized to the GAPDH. (Mean ± SD, *n* = 3, two-way ANOVA; *** *p* < 0.001).

To determine whether JEV infection alters DNAJB6 expression and to clarify the association of DNAJB6 with NS3 during JEV propagation, HEK293 cells were infected with JEV at different MOIs, and harvested at various time points. DNAJB6 mRNA levels were quantitated by qRT-PCR, and protein levels were determined by Western blotting. In HEK293 cells, DNAJB6 mRNA levels increased over the time course of JEV infection MOI =1 (Figure 7A), while DNAJB6 protein levels decreased over the time course of infection and were lower overall compared with mock-infected cells (Figure 7B). Furthermore, we found that DNAJB6 levels decreased significantly in a viral load-dependent manner (Figure 7B,C). Given these results, we reasoned that JEV infection downregulates DNAJB6 expression at post-transcriptional level in HEK293 cells.

## 3. Discussion

Some flavivirus diseases have rapidly emerged and spread globally, particularly those from flaviviruses without licensed vaccines and specific therapeutics [43]. Japanese encephalitis virus remains the leading cause of viral encephalitis in east and Southeast Asia, despite the existence of inactivated and live attenuated vaccine platforms [44]. Although the mortality risk of many flaviviruses is relatively low, the prevalence of Zika virus highlights the hazard of flaviviruses.

Considering viruses are prone to generate unpredictable mutations, interfering with steps exploited or controlled by the virus has been considered a theoretically promising approach towards antiviral therapy, albeit with limited effect up to now [45]. Pathogen-host interaction is central to the process of viral infection, thus, understanding the precise interaction between pathogen and host is essential to the development of novel vaccines and therapeutics [10]. Many of the factors required for flaviviruses entry into cells have been identified. However, we need to be aware that numerous unknowns remain, especially the host factors required for viral replication.

DnaJ/Hsp40 proteins are highly conserved throughout most of the animal kingdom, and constitute the largest and most diverse sub-group of chaperones in humans. They have diverse functions, such as participating in protein translation, folding and unfolding, translocation, and degradation, accomplished primarily by stimulating the ATPase activity of Hsp70s [46]. DnaJ/Hsp40 proteins all contain a 70 amino acid consensus sequence called the J-domain; it is this domain that interacts with Hsp70 proteins. A growing body of research on the DNAJ/Hsp40 proteins has focused on the interaction between these proteins and viral proteins. Some cellular DNAJ/Hsp40 chaperones may be beneficial for virus replication and at the same time exert antiviral activity [47]. Taguwa et al. have reported that the Dengue viral life cycle requires the Hsp70/DnaJ chaperone network [35]. Hdj2, a member of the Hsp40 family and a co-chaperone of Hsp70, directly associates with JEV NS5 to facilitate JEV replication [48]. DNAJC14 targets the yellow fever virus replication complex, inhibiting viral RNA replication, and YFV probably in turn utilizes DNAJC14′s co-chaperone function to modulate processing at the NS3/4A site to ensure virus replication [49]. DNAJC14 can also modulate the replication of bovine viral diarrhea virus. Other Flaviviridae family members, such as Kunjin, tick-borne Langat virus, and HCV, are inhibited by DNAJC14 [50]. This body of work illustrates that the members of the Flaviviridae family have evolved many pathways to interact with host Hsp40 chaperone proteins, and that the details of the Hsp40-JEV interaction still needs investigation.

Virus replication is a complex process controlled by numerous host proteins and pathways. Our study presents multiple lines of evidence demonstrating that DNAJB6, a member of Hsp40 protein family, plays a role in JEV propagation, and inhibits its replication. Expression of HA-DNAJB6 in parental HEK293 cells did suppress the mRNA levels significantly at 24 h, but had no impact at 48 h (Figure 3C and Figure 5D); the discrepancy at different time points may be because cells become diseased or exfoliated at 48 hpi. Virus replication in cells was relatively robust at 24 h; the viral titers were lower in cells overexpressing DNAJB6 at 24 hpi, although not significantly, while they were significantly decreased in cells overexpressing DNAJB6 at 48 hpi (Figure 3D). We speculate that this is because the amount of intracellular virus released into the supernatant were limited at 24 h, while at 48 h, a large number of viruses was released into the supernatant; the influence accumulated gradually and was reflected at 48 h.

Knocking out DNAJB6 in host cells resulted in a significant increase in JEV replication without alterations in virus attachment and internalization. Expressing DNAJB6 in ΔDNAJB6 cells partially restored the inhibitory effect on JEV infection (Figure 5D–F). These results demonstrate that the impairment of JEV propagation by DNAJB6 is specific. DNAJB6 protein is downregulated in response to JEV infection in a viral load-dependent manner (Figure 7B,C), indicating that JEV and DNAJB6 are mutually antagonistic and that lower levels of DNAJB6 might contribute to JEV replication.

Given the pivotal role of NS3 in JEV replication, we reasoned that the interaction between NS3 and DNAJB6 may involve modulating viral replication. This deserves further study to clarify which isoform of DNAJB6 is responsible for NS3 binding. Kampinga et al. reported that besides facilitating protein folding, several J-proteins, such as hDNAJB6 and hDNAJB8, have specific functions in preventing aggregation and shunting clients towards degradative pathways, thus maintaining aggregation-prone peptides in a form competent for peptidase degradation [42]. We speculated that DNAJB6 may bind to NS3 for degradation against virus propagation, and JEV may have evolved a way to degrade DNAJB6 to promote its replication. Our results highlight the importance of JEV NS3 in overcoming the antiviral action of DNAJB6 and offer a unique example of NS3 protein that acquires additional functions aside from those directly involved in viral replication. Together, these studies not only provide detailed insights into the replication mechanisms of JEV, but also highlight the importance of DNAJB6 as a host factor for JEV infection.

## 4. Materials and Methods

### 4.1. Cells, Virus and Replicons

Human Embryo Kidney (HEK)-293 cells (ATCC), DNAJB6 KO HEK-293 cells (generated from HEK-293 cells as parental cells in this study), HEK293T cells (ATCC), SK-N-SH cells (ATCC), and Baby Hamster Kidney cells (BHK)-21 cells (ATCC) were maintained in Dulbecco’s Modified Eagle Medium (DMEM; Gibco, Grand Island, NY, USA) supplemented with 10% fetal bovine serum (FBS; Hyclone, South Logan, UT, USA), 100 U/mL penicillin, and 100 µg/mL streptomycin. All cell lines were grown at 37 °C in a 5% CO_2_ humidified atmosphere. Cell lines were authenticated by genotyping, or obtained from authenticated stocks (ATCC).

The JEV wild-type strain used in this study was isolated from the brain tissues of aborted piglets in 2012, which belongs to genotype I (strain SCYA201201, GenBank: KM658163) [51]. The subgenomic replicon, SA14/U14163-Replicon (Rluc-rep), was a kind gift from Dr. Bo Zhang.

### 4.2. Plasmids and Antibodies

The plasmids and antibodies used in this study are listed in Table 2. The full length NS3 gene was amplified using the sequence of SCYA201201 strain (GenBank: KM658163) as a template; the bait plasmid used for yeast-two hybrid experiments, pGBKT7-NS3, was constructed by inserting the NS3 gene into the pGBKT7 vector. pEGFP-NS3, used for Co-IP, was constructed by inserting the full-length PCR amplified NS3 gene into the pEGFP-N1 vector. The cDNA of human DNAJB6 (NCBI accession number NM_005494.2) was amplified from HEK293 cells by PCR. The PCR product was digested with EcoRI and XhoI, and inserted into the pCMV-HA vector. The HA-tagged DNAJB6 expression plasmid was used for Co-IP and overexpression. The primers used for plasmids construction in this study are listed in Table 3. Sequences of all the recombinant plasmids were verified by DNA sequencing.

### 4.3. Yeast Two-Hybrid Screening

The Matchmaker Gold Yeast Two-Hybrid system (Clontech, Mountain View, CA, USA) was used to screen for host proteins that interact with NS3. The bait yeast was generated by transforming the Y2HGold strain with the pGBKT7-NS3 bait plasmid. The strain was tested for toxicity, autoactivation, and target protein expression. The bait yeast was mated with a human brain tissue cDNA library constructed using strain Y187 (Clontech, Mountain View, CA, USA) containing inserts cloned into the pGADT7-Rec (AD) vector, following the manufacturer’s instructions. Heterozygotes were plated on double-dropout medium (minimal, synthetic defined medium for yeast (SD), lacking leucine and tryptophan). The clones were then plated on quadruple-dropout medium (QDO/X/Aba, SD lacking adenine, histidine, leucine, and tryptophan, but containing X-alpha-galactosidase and aureobasidin A). After three rounds of screening, positive clones were acquired. The yeast plasmids were extracted, and the prey plasmids were isolated by transformation into *Escherichia coli* DH5α with selection using ampicillin. The prey plasmids were sequenced, and the sequences were aligned against the human non-RefSeq databases at NCBI using the web-based Basic Local Alignment Search Tool (BLASTN), the analysis was conducted using the default parameters. To confirm the interaction between NS3 and the cellular proteins, Y2HGold was co-transformed with the prey plasmid AD and the bait plasmid BD-NS3 or BD. The transformants were grown on DDO and QDO/X-α-Gal/Aba plates. Co-transformation with BD-p53/AD-T (simian virus 40 [SV40] large T antigen), BD-Lam (human lamin C protein)/AD-T, and BD/AD served as positive, negative, and blank controls, respectively.

### 4.4. Transfections

DMRIE-C Reagent (Invitrogen, Carlsbad, CA, USA) was used for transfecting cells with RNA; transfections were done in six-well plates. Lipofectamine 2000 (Invitrogen, Carlsbad, CA, USA) was used for transfecting HEK293 cells with plasmid DNA; transfections were done in 60-mm dishes. All procedures were performed according to the manufacturer’s recommendations.

### 4.5. Co-immunoprecipitation Assays

Co-immunoprecipitation was performed as follows: 10^7^ HEK293 cells were transfected with the indicated plasmids; at 48 h post-transfection, the cells were harvested and lysed with Native Lysis Buffer containing protease inhibitor cocktail. The total cell lysate was precleared with Protein A magnetic beads. Samples were then incubated with the relevant antibodies overnight at 4 °C with gentle shaking. Protein A magnetic beads were added, and the samples were incubated for another 3 h at room temperature with gentle shaking. The magnetic beads were subsequently washed three or more times with wash buffer. The bound proteins were eluted by boiling with SDS-PAGE loading buffer for 5 min and then subjected to Western blotting.

### 4.6. Virus Infection Assays

JEV, at the indicated multiplicity of infection (MOI), was adsorbed to monolayers of HEK293 cells for 1 h at 37 °C. Unbound virus was removed by washing with serum-free DMEM; then, cells were overlaid with, and maintained in, Dulbecco’s modified Eagle’s medium with 2% fetal bovine serum at 37 °C. Viral replication was measured by plaque assay on BHK-21 cells.

### 4.7. Confocal Immunofluorescence Microscopy

For colocalization studies, HEK293 cells, grown on coverslips in 12-well plates, were transfected with the DNAJB6-HA plasmid, followed by infection with JEV SCYA201201 at an MOI of 1.0. At 36 h post-infection, cells were fixed with 4% paraformaldehyde for 30 min, followed by washing with PBS, and permeabilization with 0.1% Triton X-100 for 30 min at room temperature. Cells were then incubated with the appropriate primary antibodies overnight at 4 °C. After washing, the cells were incubated with a 1:200 dilution of Alexa Fluor 488- and Alexa Fluor 555-conjugated secondary antibodies for 1 h, then stained with DAPI for another 10 min. Images were acquired on an Olympus FV3000 confocal microscope. Z-stacks were acquired by sequential scanning.

### 4.8. Generation of DNAJB6 Knock-Out Cell Lines and DNAJB6 Complementation with Exogenous Plasmid

We used the CRISPR/Cas9 system to generate DNAJB6 knock-out HEK293 clones. In brief, using Lipofectamine 2000 (Invitrogen), HEK293 cells were transfected with pSpCas9(BB)-2A-GFP (PX458) (Addgene plasmid 48138) harboring a guide RNA (gRNA) targeting exon 6 (5′- GGGAATCGAAGGGGTCCCCG-3′), followed by subclone selection. Positive transfected cells were selected, DNAJB6 deficiency in cell lines was confirmed by Sanger sequencing, and clonal cells were analyzed for DNAJB6 KO by Western blot. Next, ΔDNAJB6 cells was transfected with the pCMV-HA-DNAJB6 plasmid to evaluate the effect of trans-complementation of DNAJB6 on JEV propagation in ΔDNAJB6 cells, which are common necessary steps to form a close cycle.

### 4.9. Quantitative RT-PCR

Cells were treated or infected as indicated; then, total RNA was extracted from the cells using TRIzol Reagent. Reverse transcription was performed with PrimeScript RT Reagent Kit with gDNA Eraser (TaKaRa, Tokyo, Japan). The resulting cDNA was used for quantitative real-time PCR with specific primers (Table 4), using SYBR green I Premix (TaKaRa, Tokyo, Japan) in a CFX96™ Real-Time PCR Detection System. Relative mRNA values were calculated using the 2^−ΔΔCT^ method with the level of β-actin expression in each sample as an internal control and shown as fold change by normalizing to the mock-control. Each experiment contained three biological duplicates, and qPCR for each sample was done in triplicate. PCR conditions for the JEV E gene (accession number KM658163) amplification were as follows: 30 s at 95 °C, followed by 40 cycles of 95 °C for 5 s, 54 °C for 30 s, and 72 °C for 30 s. PCR conditions for the DNAJB6 (accession number NM_005494) amplification were: 30 s at 95 °C, followed by 40 cycles of 95 °C for 5 s, 52 °C for 30 s, and 72 °C for 1 min. Further details of the RT and PCR conditions are available upon request.

### 4.10. Immunoblotting

Total cell lysates were prepared using radioimmunoprecipitation assay buffer (Sigma-Aldrich, St. Louis, MO, USA) containing protease inhibitors (Roche, Bassel, Switzerland). Protein concentrations were determined using a BCA protein assay kit (Beyotime, Shanghai, China). Equal quantities of protein were separated by SDS-PAGE then transferred to a polyvinylidene fluoride membrane (Bio-Rad, Hercules, CA, USA) using a Trans-Blot Semi-Dry Transfer apparatus (Bio-Rad, Hercules, CA, USA). Glyceraldehyde-3-phosphate dehydrogenase (GAPDH) was used as an internal control. Blots were probed with the relevant antibodies, and proteins were detected using enhanced chemiluminescence reagent (Bio-Rad, Hercules, CA, USA).

### 4.11. Plaque Assay

Infected cell supernatants were titered using a plaque forming assay. BHK-21 cells were seeded in six-well plates and incubated until they formed monolayers (1–2 days). Supernatants were serially diluted then inoculated onto BHK-21 cells. After 1.5 h incubation at 37 °C, the cells were washed with serum-free DMEM and cultured in DMEM containing 2% fetal bovine serum and 1.25% sodium carboxymethyl cellulose for 4 days. The cells were then stained with crystal violet, and fixed with formaldehyde for 30 min at room temperature. Visible plaques were counted, and the viral titers were calculated. All data are expressed as means of triplicate samples.

### 4.12. Cell Viability Assays

A CellTiter-Lumi™ Luminescent Cell Viability Assay Kit (Beyotime, Shanghai, China) was used according to the manufacturer’s instructions. In brief, equal numbers of ΔDNAJB6 cells and parental HEK293 cells in 100 μL of culture medium were seeded into opaque-walled 96-well plates and incubated for 24 h. 100 μL of Cell Titer-Luminescent reagent was added to each well and plates were shaken for 2 min. After a 10 min incubation at room temperature, luminescence was recorded using a Varioskan Flash (Thermo Fisher Scientific, Waltham, MA, USA) with an integration time of 0.5 s per well. The generation of luminescent signal is proportional to the amount of ATP present.

### 4.13. Viral Binding and Internalization Assays

ΔDNAJB6 and parental HEK293 cells were incubated with JEV at a MOI of 10 for 1 h at 4 °C, then washed to remove unbound virus. The amount of bound virus was quantitated by RT-qPCR. To determine the amount of internalized virus, the cells with bound virus were washed as above, overlaid with warm media and incubated for 1 h at 37 °C. After incubation, the cells were washed with chilled PBS, followed by proteinase K treatment (1 mg/mL) for 45 min at 4 °C to remove the membrane-bound virions that had not been internalized. The cells were then lysed, and RNA was extracted using TRIzol reagent; the endocytosed JEV RNA was quantitated using RT-qPCR, as described previously.

### 4.14. In Vitro RNA Transcription, Transfection, and Luciferase Assays

The plasmids carrying the JEV subgenomic replicon fused with a luciferase reporter gene were linearized by Xhol, purified by phenol-chloroform extraction, then transcribed in vitro using the mMESSAGEmMACHINE^®^ T7 Kit (Ambion) according to the manufacturer’s instructions. Then, 2 μg of the RNA was transfected into 10^6^ ΔDNAJB6 or parental cells per well using the DMRIE-C Reagent (Invitrogen, Carlsbad, CA, USA). At different times post-transfection, the cells were harvested and the replication of the JEV replicon was analyzed by luciferase assay and Western blotting. Luciferase activity was measured in a microplate reader (Varioskan Flash; Thermo Fisher Scientific, Waltham, MA, USA) by mixing 20 μL lysate with 100 μL substrate (Promega, Medison WI, USA).

### 4.15. Quantification and Statistical Analysis

All data are expressed as means ± standard deviation (SD) as indicated. Student’s unpaired t test was used to estimate the statistical significance between two groups, whereas ANOVA was used to compare the means among three or more groups. When there is one categorical independent variable, statistical significance was analyzed with a one-way ANOVA, whereas for two categorical independent variables, statistical significance was analyzed with a two-way ANOVA. Test of normality and test of homogeneity of variances were performed by using SPSS software (Version 23.0, IBM, Armonk, NY, USA) are before the corresponding statistical analysis applied. The statistical data (*n*-numbers, mean ± SD and tests) are given in the figure legends and a *p* value of 0.05 was considered statistically significant. Statistical significance is indicated as * (*p* < 0.05), ** (*p* < 0.01), *** (*p* < 0.001) and ns (not significant). All statistical analyses and calculations were performed using GraphPad Prism (Version 6, La Jolla, CA, USA).

## Figures and Tables

**Table 1:** BLAST results for clones obtained from the yeast two-hybrid screen.

Protein No.	Protein Name	Gene	NCBI Protein Accession Number	Max Identity (%)	Number of Clones
1	COP9 constitutive photomorphogenic homolog subunit 5 (Arabidopsis) [Homo sapiens]	COPS5	AAH01859.1	99%	6
2	fibulin-5 precursor [Homo sapiens]	FBLN5	NP_006320.2	100%	1
3	serine/threonine-protein phosphatase 2A catalytic subunit beta isoform [Homo sapiens]	PPP2CB	NP_001009552.1	100%	1
4	Cereblon [Homo sapiens]	CRBN	AAH17419.1	99%	1
5	dnaJ homolog subfamily B member 6 isoform b [Homo sapiens]	DNAJB6	NP_005485.1	100%	4
6	ubiquitin-conjugating enzyme E2 N [Homo sapiens]	UBE2N	NP_003339.1	99%	1
7	zinc finger protein 350 [Homo sapiens]	ZNF350	NP_067645.3	100%	1
8	integral membrane protein GPR137B [Homo sapiens]	GPR137B	NP_003263.1	100%	1

**Table 2:** Plasmids and antibodies used in this study.

Plasmid and Antibody	Source	Identifier
pGBKT7-NS3	Research Center of Swine Disease	N/A
pCMV-HA-DNAJB6	Research Center of Swine Disease	N/A
pEGFP-NS3	Research Center of Swine Disease	N/A
SA14/U14163 replicon (Rluc-rep)	[52]	N/A
Anti-DNAJB6 antibody [EPR17122]-N-terminal	Abcam	Cat#ab198995
GAPDH polyclonal antibody	ABclonal Technology	Cat#AC001
Monoclonal anti-HA antibody	Sigma Aldrich	Cat#H9658
Alexa Fluor 488-conjugated goat anti-rabbit IgG	Beyotime	Cat#A0423
Alexa Fluor 555-conjugated donkey anti-mouse IgG	Beyotime	Cat#A0460
Mouse and rabbit polyclonal antibodies against JEV Anti-NS3 antibodies	Research Center of Swine Disease	N/A

**Table 3:** Primers used in plasmids construction.

Plasmid	Primer Name	Sequence (5′–3′)
pGBKT7-NS3	pGBKT7-NS3-F	CGGGATTCGGGGGCGTGTTCTGGGACACG
	pGBKT7-NS3-R	CGCGTCGACTCTCTTGCCCGCTGCAAAATCC
pEGFP-NS3	pEGFP-NS3-F	CGAGCTCGCCACCATGGGGGGCGTGTTCTGGGACACG
	pEGFP-NS3-R	TCCCCGCGGTCTCTTGCCCGCTGCAAAATCC
pCMV-HA-DNAJB6	HA-DNAJB6-F	CTGAATTCGGATGGTGGATTACTATGAAGTTCTAG
	HA-DNAJB6-R	TGCTCGAGTTACTTGTTATCCAAGCGCAGCA

**Table 4:** Primers used in qRT-PCR.

Primer Name	Sequence (5′–3′)
JEV-E-F	CAGTGGAGCCACTTGGGTG
JEV-E-R	TTGTGAGCTTCTCCTGTCG
DNAJB6-F	ATGCTAAGAAACGGGACA
DNAJB6-R	ATCTGGGTTACGGAATG
β-actin-F	GTGGACATCCGCAAAGAC
β-actin-R	AAAGGGTGTAACGCAACTA

## Supplementary Materials

Supplementary materials can be found at https://www.mdpi.com/1422-0067/20/22/5719/s1, Figure S1: Immunofluorescence and western blot were performed to verify antibody specificity.
