# Supplementary material for: Hsp40 Protein DNAJB6 Interacts with Viral NS3 and Inhibits the Replication of the Japanese Encephalitis Virus
Source: Int J Mol Sci. 2019 Nov 14;20(22):5719. doi: 10.3390/ijms20225719 (PMC6888364; doi:10.3390/ijms20225719)
Supplement: Supplementary file 1 [file ijms-20-05719-s001.pdf]

Hsp40 Protein DNAJB6 Interacts with Viral NS3 and Inhibits the Replication of the Japanese Encephalitis Virus

Yu-Qin Cao , Lei Yuan , Qin Zhao , Jian-Lin Yuan , Chang Miao , Yung-Fu Chang , Xin-Tian Wen , Rui Wu , Xiao-Bo Huang , Yi-Ping Wen , Qi-Gui Yan , Yong Huang , Xin-Feng Han, Xiao-Ping Ma and San-Jie Cao

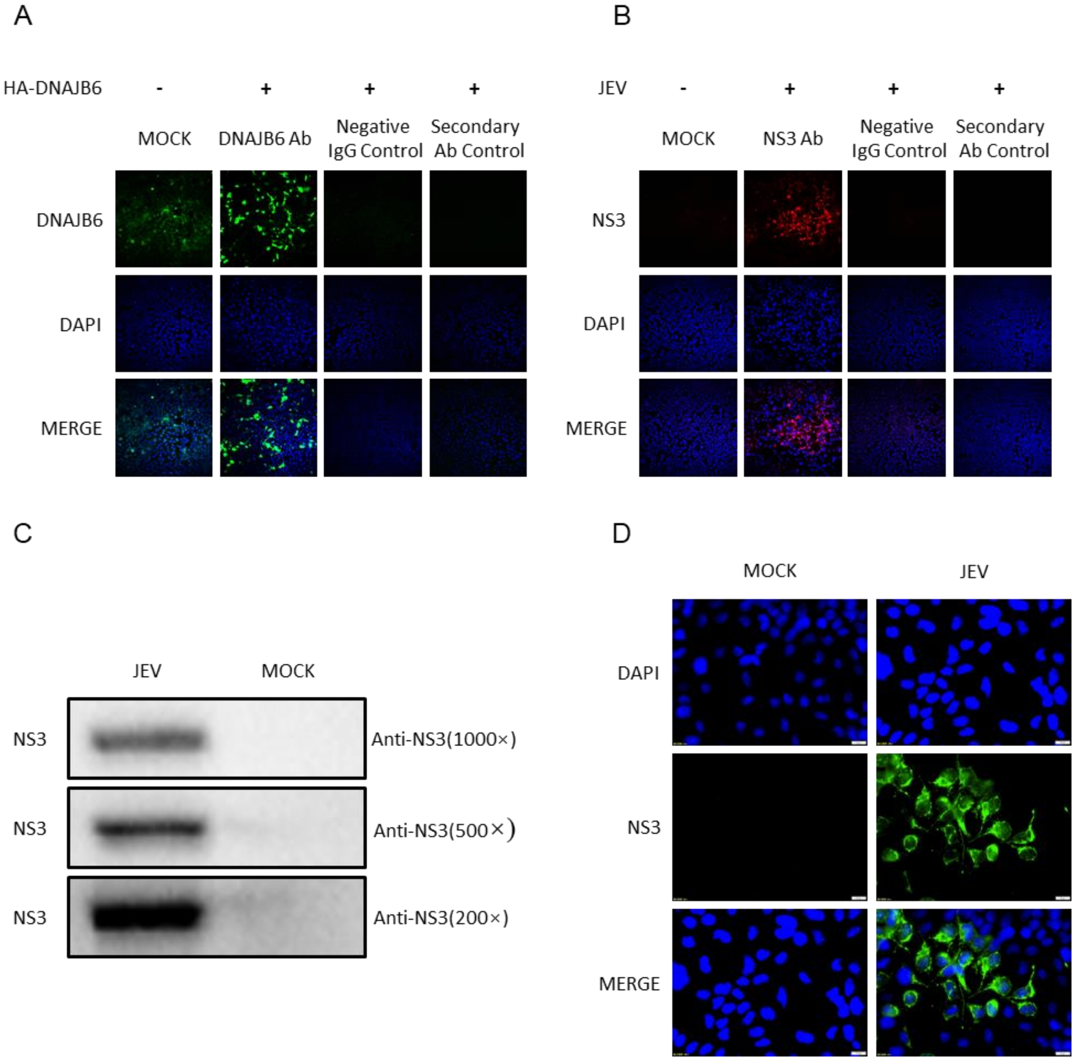

Figure S1. Immunofluorescence and western blot were performed to verify antibody specificity. **(A)** Immunofluorescence assays were performed to detect anti-DNAJB6 rabbit antibody specificity using a 1:200 dilution of the antibody. **(B)** Immunofluorescence assays were performed to detect anti-NS3 mouse antibody specificity using a 1:200 dilution of the antibody. **(C)** Western blot was performed to detect anti-NS3 rabbit antibody specificity and sensitivity using dilutions of 1:200, 1:500, and 1:1000 dilutions. **(D)** Immunofluorescence assays were performed to detect anti-NS3 rabbit antibody sensitivity using a 1:200 dilution of the antibody.
